# Febrile-range temperature selectively limits sustained pDC-Derived IFN-α via attenuation of STAT1-Dependent feedback signaling

**DOI:** 10.1016/j.cellin.2026.100344

**Published:** 2026-06-24

**Authors:** Yangyang Xu, Hao Yang, Lin Yang, Qing Zhang, Liang Cheng

**Affiliations:** a Department of Infectious Diseases, Medical Research Institute, Frontier Science Center for Immunology and Metabolism, State Key Laboratory of Virology and Biosafety, Zhongnan Hospital of Wuhan University, Wuhan University, Wuhan, 430071, Hubei, China; b Department of Otolaryngology, Head and Neck Surgery, Zhongnan Hospital of Wuhan University, Wuhan University, Wuhan, 430071, Hubei, China; c Department of General Surgery, General Hospital of Xuzhou Mining Group, Xuzhou, 221000, Jiangsu, China; d Cancer Institute, Xuzhou Medical University, Xuzhou, 221004, Jiangsu, China

**Keywords:** pDC, Interferon-α, Febrile temperature, STAT1 phosphorylation

## Abstract

Plasmacytoid dendritic cells (pDCs) are specialized sentinels of antiviral immunity and the dominant early source of interferon-α (IFN-α) during viral infection. While pDC-derived IFN-α restricts viral replication and provides a critical third signal for T cell activation, sustained type I interferon (IFN-I) signaling can drive immunopathology, and the physiological mechanisms that constrain this response remain incompletely understood. Here, we identify fever as a conserved host factor that selectively limits IFN-α production by pDCs. We show that febrile-range temperature does not impair early IFN-α induction but instead suppresses sustained IFN-α output in human and mouse pDCs. Mechanistically, elevated temperature attenuates signal transducer and activator of transcription 1 (STAT1) phosphorylation, thereby disrupting the IFN-I–dependent positive feedback loop required for IFN-I amplification following CpG-A stimulation. Pharmacological inhibition of protein phosphatase activity with okadaic acid (OA) restores STAT1 phosphorylation and rescues IFN-α production under febrile conditions. Together, these findings establish fever as a physiological regulator of pDC-derived IFN-I responses and reveal a temperature-dependent mechanism that constrains prolonged IFN-I production to prevent excessive immune activation.

## Highlights

• Fever acts as a physiological brake on pDC-derived type I interferon responses.
• Elevated temperature uncouples early IFN-I induction from STAT1-dependent amplification.
• Thermal attenuation of the IFN-I feedback loop prevents excessive interferon output.

## Graphical abstract

**Figure:**
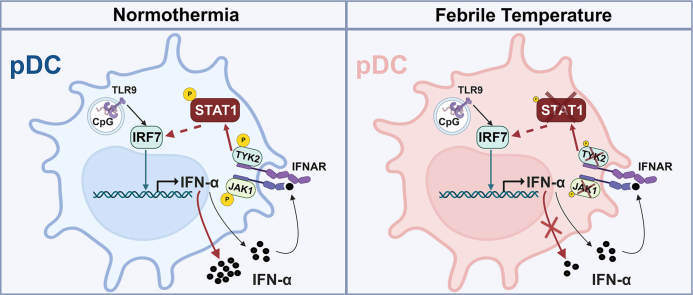

## 1 Introduction

Fever is a conserved physiological response to infection and tissue injury that has evolved to enhance host survival across ectothermic and endothermic species (Earn et al., 2014; Kluger, 1986; Schulman et al., 2005). Clinical evidence supports a protective role for fever during infection, as antipyretic treatment has been associated with increased mortality in severe disease (Schulman et al., 2005).

Beyond its antimicrobial effects, fever functions as a systemic danger signal that shapes immune cell behavior. By elevating core body temperature, fever exerts direct inhibitory effects on microbial growth while simultaneously modulating immune responses to promote pathogen clearance (Evans et al., 2015). Elevated temperature has been shown to regulate the recruitment, activation, and effector functions of multiple immune cell types, including neutrophils, natural killer cells, macrophages, dendritic cells (DCs), and lymphocytes (Evans et al., 2015; Kappel et al., 1991; Ostberg et al., 2000; Pritchard et al., 2005; Rice et al., 2005). Fever enhances innate immune responses by promoting inflammatory signaling pathways and cytotoxic activity, while also influencing adaptive immunity through effects on antigen presentation, lymphocyte trafficking, and T cell differentiation (Basu and Srivastava, 2003; Lin et al., 2019; Lee et al., 2020). Notably, fever can also exert immunopathogenic effects, as exemplified by its ability to promote pathogenic T helper 17 (Th17) differentiation in autoimmune settings, highlighting the need for precise physiological control of immune activation (Wang et al., 2020).

Plasmacytoid DCs (pDCs) are a rare subset of peripheral blood mononuclear cells that selectively express Toll-like receptors TLR9 and TLR7 and are uniquely specialized for the rapid production of type I interferons (IFN-I) (Siegal et al., 1999; Adams et al., 2024). Upon viral or nucleic acid stimulation, pDCs produce IFN-I at levels orders of magnitude higher than other immune cells, thereby playing a central role in initiating antiviral immunity (Gilliet et al., 2008; Yu et al., 2011). pDC-derived IFN-I induces interferon-stimulated genes, promotes apoptosis of infected cells, and establishes an antiviral state (Swiecki and Colonna, 2015; Reizis, 2019). Our group and others have recently found that dysregulated IFN-α production by human and mouse pDCs, however, can contribute to viral persistence, chronic infection, or immunopathology, underscoring the importance of mechanisms that constrain IFN-I responses (Cheng et al., 2017; Li et al., 2014, 2025; Ngo et al., 2025; Su et al., 2026; Zhao et al., 2018).

While fever is known to regulate multiple immune cell types, its impact on pDC function and IFN-I production remains poorly understood. In this study, we demonstrate that febrile temperature selectively suppressed sustained IFN-I production by human and mouse pDCs without impairing early IFN-α induction. Mechanistically, elevated temperature disrupted the STAT1-dependent positive feedback loop required for IFN-I amplification, thereby limiting prolonged IFN-α output. Importantly, pharmacological restoration of STAT1 phosphorylation rescued IFN-α production under febrile conditions. These findings identified fever as a physiological regulator of pDC-derived IFN-I responses and reveal a previously unrecognized mechanism by which elevated temperature constrains antiviral immunity to prevent immune overactivation.

## 2 Results

### 2.1 Febrile temperature selectively suppresses TLR9-mediated IFN-α production by pDCs

**Fig. 1:**
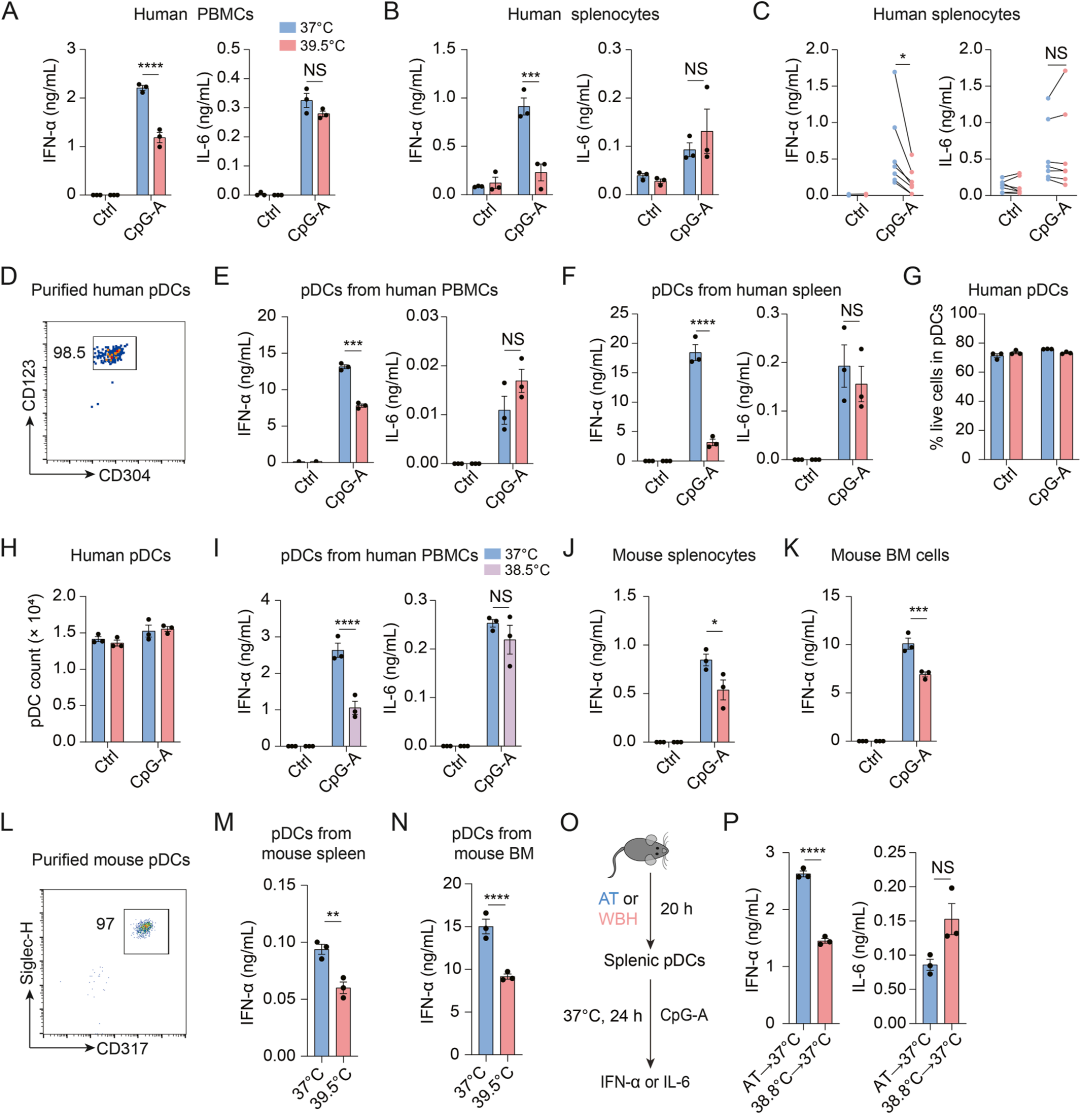
**Febrile temperature suppresses IFN-α production by pDCs from humans and mice.** Human peripheral blood mononuclear cells (PBMCs) (A), human splenocytes (B and C), purified human pDCs (D-I), mouse splenocytes (J), mouse bone marrow (BM) cells (K), and purified mouse pDCs (L-N) were used in the indicated experiments. Cells were pre-incubated at 37 °C, 38.5 °C, or 39.5 °C for 1 h, followed by stimulation with 1 μM CpG-A for 24 h. Supernatants or cells were collected for subsequent analyses. (A–C) Human IFN-α and IL-6 levels in culture supernatants measured by ELISA. (D) Purity of sorted human pDCs from spleen assessed by flow cytometry. (E, F) IFN-α and IL-6 levels in culture supernatants of purified human pDCs measured by ELISA. (G) Proportion of viable human pDCs after 24 h of activation at the indicated temperatures, assessed by flow cytometry. (H) Absolute numbers of viable human pDCs after 24 h of activation at the indicated temperatures. (I) IFN-α and IL-6 levels in culture supernatants of purified human pDCs measured by ELISA. (J, K) Mouse IFN-α levels in culture supernatants of splenocytes (J) and BM cells (K) measured by ELISA. (L) Purity of purified mouse pDCs assessed by flow cytometry. (M, N) IFN-α levels in culture supernatants of purified mouse pDCs measured by ELISA. (O) Mice were maintained at ambient temperature (AT) or subjected to fever-range whole-body hyperthermia (WBH) for 20 h prior to sacrifice. pDCs were purified from spleens and stimulated *ex vivo* with 1 μM CpG-A at 37 °C for 24 h. (P) IFN-α and IL-6 levels in culture supernatants measured by ELISA. The IFN-α levels in (A, F, I, J, and K) and the IL-6 levels in (E, F, and I) for the control groups were below the ELISA limit of detection. Data in panels (A), (B), (D), (E), (F), (G), (H), and I are representative of one donor from at least three independent donors. Data in panel (C) represent pooled results from seven donors. Experiments shown in panels (J–P) were repeated at least three times. Data are presented as mean ± SEM (A, B, E–K, M, N, and P) or as individual values (C). Statistical significance was determined using two-way ANOVA (A, B, C, E–K) and unpaired Student's *t*-test (M, N, and P). ∗*p* < 0.05, ∗∗*p* < 0.01, ∗∗∗*p* < 0.001, ∗∗∗∗*p* < 0.000 1; NS, not significant.

To investigate the effects of febrile temperature on IFN-α production, human peripheral blood mononuclear cells (PBMCs) were cultured under normothermic (37 °C) or febrile (39.5 °C) conditions in the presence of TLR9 agonist CpG-A. IFN-α production in supernatant was quantified after 24 h. CpG-A stimulation induced substantial IFN-α production at 37 °C (2 208.9 ± 49.1 pg/mL), which was significantly reduced at 39.5 °C (1 183.5 ± 104.3 pg/mL) (Fig. 1(A)). In contrast, IL-6 production remained comparable between temperature conditions (Fig. 1(A)). Similar results were observed by utilizing human splenocytes (Fig. 1(B)), and the findings were consistent across 7 more different donors (Fig. 1(C)). Furthermore, increasing temperature progressively suppressed IFN-α production in human PBMCs (Fig. S1). Because TLR9 expression in human PBMCs is largely restricted to pDCs and B cells (Kadowaki et al., 2001; Hornung et al., 2002), and pDCs are the predominant producers of type I interferons in response to CpG-A stimulation (Krug et al., 2001), these data suggest that fever-range temperature selectively impaired IFN-I production while preserving IL-6 secretion in human pDCs.

To further confirm pDC-specific effects, we purified CD123^+^CD304^+^ pDCs (Fig. 1(D)) from both human peripheral blood and spleen tissues and stimulated them with CpG-A at varying temperature. 39.5 °C reproducibly suppressed IFN-α production in purified pDCs from either peripheral blood (41% reduction vs. 37 °C) or spleens (83% reduction vs. 37 °C), while maintaining IL-6 output (Fig. 1(E) and (F)). Cell viability assays confirmed no significant differences in viability (Fig. 1(G)) or live cells recovered (Fig. 1(H)) between different culture conditions. At 38.5 °C, pDCs similarly lowered IFN-α production by 60%, while the production of IL-6 remained unchanged (Fig. 1(I)).

This thermal regulation of IFN-α production was evolutionarily conserved, as parallel experiments with murine splenocytes or bone marrow cells (Fig. 1(J) and (K)), as well as purified CD317^+^Siglec-H^+^ pDCs from murine spleens or bone marrow (Fig. 1(L–N)) also demonstrated that 39.5 °C culture condition significantly inhibit mouse IFN-α production. To study whether febrile temperature affects pDCs *in vivo*, we utilized the well-recognized mouse model whole-body hyperthermia (WBH) (Lin et al., 2019; Lin et al., 2021; Chen et al., 2006). Mice were exposed to either ambient temperature (AT) or fever-range WBH for 20 h prior to sacrifice and pDCs were isolated from the spleens and subsequently stimulated with CpG-A at 37 °C *ex vivo* for 24 h (Fig. 1(O)). The results showed that pDCs isolated from mice with WBH produced lower levels of IFN-α (45% reduction), with the production of IL-6 unchanged (Fig. 1(P)).

Collectively, these findings demonstrate that elevated physiological temperature specifically suppress TLR9-mediated IFN-α production by pDCs.

### 2.2 Febrile temperature reprograms the transcriptome of CpG-A–activated pDCs and suppresses IFN-I–associated signaling

**Fig. 2:**
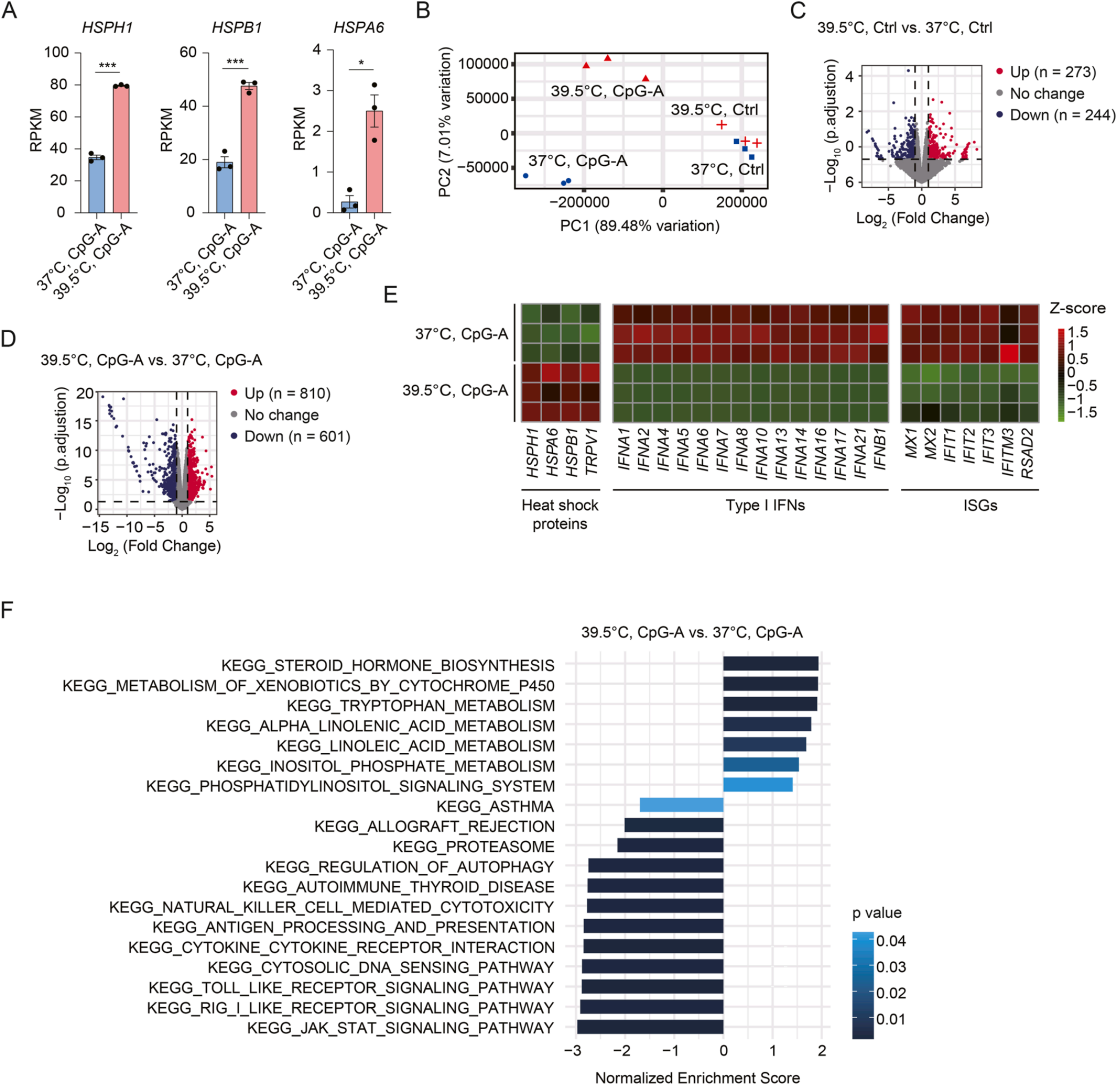
**Febrile temperature reprograms the transcriptome of CpG-A–Activated pDCs and suppresses IFN-I–associated signaling.** Human pDCs purified from spleen were stimulated with or without CpG-A (1 μM) for 24 h under normothermic (37 °C) or febrile (39.5 °C) conditions. Cells were then harvested for RNA-seq analysis. (A) Expression of the indicated heat shock protein genes in pDCs cultured at 37 °C or 39.5 °C. (B) Principal component analysis (PCA) of pDC transcriptomes under each condition (*n* = 3 per group). (C, D) Volcano plots showing differentially expressed genes in pDCs cultured at different temperatures in the absence of stimulation (C) or following CpG-A stimulation (D). Differentially expressed genes were defined as *p* < 0.05 with |log_2_FC| ≥ 1. (E) Heatmap displaying the expression of selected genes across conditions. (F) GSEA of the indicated pathways in CpG-A–stimulated pDCs cultured at 39.5 °C compared with those cultured at 37 °C.

To characterize the transcriptomic changes in pDCs under normothermic (37 °C) or febrile (39.5 °C) conditions with or without CpG-A stimulation, we performed RNA sequencing at 24 h post-stimulation. The expected induction of heat shock protein genes at febrile temperature validated the experimental system (Fig. 2(A); Table S1). Principal component analysis (PCA) showed that unstimulated pDCs cultured at febrile temperature clustered closely with their normothermic counterparts (Fig. 2(B)). In contrast, CpG-A–stimulated pDCs cultured under febrile conditions were clearly separated from normothermic-stimulated cells along both PC1 and PC2, indicating substantial transcriptomic reprogramming induced by elevated temperature (Fig. 2(B)). Detailed analysis showed that pDCs cultured at 39.5 °C in the absence of stimulation exhibited upregulation of 273 genes and downregulation of 244 genes compared with unstimulated normothermic controls (Fig. 2(C); Table S1). Upon CpG-A stimulation, pDCs cultured at 39.5 °C displayed more pronounced transcriptional alterations, with 810 genes upregulated and 601 genes downregulated relative to CpG-A–stimulated pDCs under normothermic conditions (Fig. 2(D); Table S1).

Consistent with protein expression detected by enzyme-linked immunosorbent assay (ELISA), single-gene expression analysis revealed coordinated suppression of all 13 *IFNA* subtypes, *IFNB1*, and interferon-stimulated genes (ISGs) in febrile CpG-A–stimulated pDCs (Fig. 2(E)), whereas *IL6* expression remained unchanged (Table S1). Gene set enrichment analysis (GSEA) further confirmed significant suppression of toll-like receptor signaling, cytosolic DNA sensing, antigen processing and presentation, JAK–STAT signaling pathways in febrile CpG-A–stimulated pDCs (Fig. 2(F)). Conversely, metabolic pathways such as steroid hormone biosynthesis, tryptophan metabolism, and inositol phosphate metabolism were enriched under febrile conditions (Fig. 2(F)).

Collectively, these data demonstrate that febrile temperature profoundly impairs IFN-I transcription and disrupts downstream antiviral signaling programs in CpG-A–activated pDCs.

### 2.3 Thermosensory and metabolic pathways do not account for febrile temperature–mediated suppression of IFN-α production

**Fig. 3:**
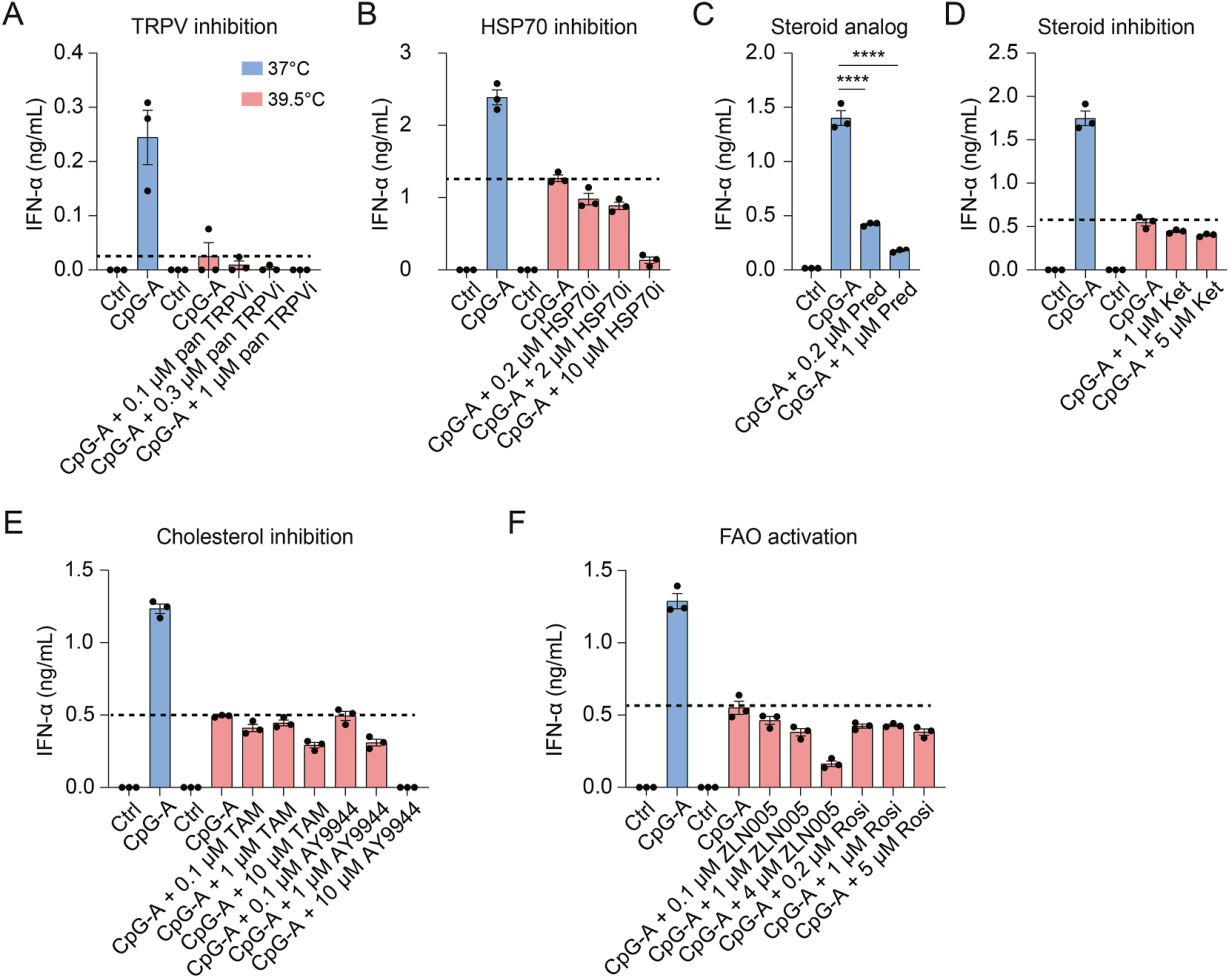
**Thermosensory and metabolic pathways do not account for febrile temperature–mediated suppression of IFN-α production.** Human PBMCs (B) or splenocytes (A, C–F) were stimulated with or without CpG-A (1 μM) at 37 °C or 39.5 °C for 24 h in the presence or absence of the indicated inhibitors. IFN-α levels in culture supernatants were quantified by ELISA. (A) IFN-α secretion by human splenocytes cultured under the indicated conditions in the presence of increasing concentrations of a TRPV channel inhibitor. (B) IFN-α secretion by human PBMCs cultured under the indicated conditions in the presence of increasing concentrations of an HSP70 inhibitor. (C) IFN-α secretion by human splenocytes cultured under the indicated conditions in the presence of increasing concentrations of the synthetic glucocorticoid prednisolone (Pred). (D) IFN-α secretion by human splenocytes cultured under the indicated conditions in the presence of increasing concentrations of the steroid synthesis inhibitor ketoconazole (Ket). (E) IFN-α secretion by human splenocytes cultured under the indicated conditions in the presence of increasing concentrations of the cholesterol biosynthesis inhibitors tamoxifen (TAM) or AY9944. (F) IFN-α secretion by human splenocytes cultured under the indicated conditions in the presence of modulators of fatty acid oxidation (FAO), including the PGC-1α agonist ZLN005, and the PPARγ agonist rosiglitazone (Rosi). The control group IFN-α levels in (A–F) were below the ELISA limit of detection. Data in are representative of one donor from 3 donors, presented as mean ± SEM.

Based on the transcriptomic changes observed under febrile conditions, we next asked whether temperature-induced metabolic and stress-response pathways mechanistically contribute to the suppression of IFN-α production in pDCs. To address this, we first examined whether thermosensory signaling pathways are required for the febrile temperature–mediated attenuation of IFN-α output. Pharmacological inhibition of transient receptor potential vanilloid (TRPV) channels failed to rescue IFN-α production in primary splenocytes exposed to febrile temperature (Fig. 3(A)).

The inhibition of heat shock protein 70 (HSP70) has been reported to inhibit Th17 differentiation under febrile conditions (Wang et al., 2020). Given the temperature-sensitive induction of heat shock proteins (HSPs) under febrile conditions (Fig. 2(A)), we next assessed the contribution of HSP response. The results showed that treatment with an HSP70 inhibitor at low concentrations (0.2 and 2 μM) did not affect IFN-α production by CpG-A-stimulated PBMCs under febrile temperature, while high concentration (10 μM) reduced IFN-a production (Fig. 3(B)). Importantly, none of the concentrations tested significantly affected pDC viability at febrile temperature (Fig. S2).

Steroid biosynthesis emerged as one of the most prominently upregulated metabolic pathways in GSEA analysis (Fig. 2(F)). Consistent with previous reports that steroids suppress IFN-α production in pDCs (Boor et al., 2006), we confirmed that treatment of splenocytes with the steroid analog prednisolone (Pred) markedly reduced IFN-α production (Fig. 3(C)). However, inhibition of steroid synthesis using ketoconazole (Ket) failed to restore IFN-α production under febrile conditions (Fig. 3(D)). Furthermore, although inhibition of the terminal cholesterol biosynthesis enzyme 7-dehydrocholesterol reductase (DHCR7) has been shown to enhance IFN-I production in macrophages under normothermic condition (Wang et al., 2023), pharmacological blockade of DHCR7 using tamoxifen (TAM) or AY9944 did not reverse the thermal suppression of IFN-I production (Fig. 3(E)).

Previous studies have demonstrated a role for fatty acid oxidation (FAO) metabolism in regulating pDC activation (Wu et al., 2016). However, activation of FAO signaling using PGC-1α agonist ZLN005, or PPARγ agonist rosiglitazone (Rosi) did not rescue IFN-α production in human splenocytes cultured at febrile temperature (Fig. 3(F)).

Together, these results indicate that inhibition of thermosensory pathways, heat shock responses, steroid biosynthesis, or activation of FAO is insufficient to restore IFN-α production in pDCs under febrile conditions, suggesting that febrile temperature suppresses pDC function through alternative regulatory mechanisms.

### 2.4 Febrile temperature suppresses IFN-α production through attenuation of STAT1 phosphorylation

**Fig. 4:**
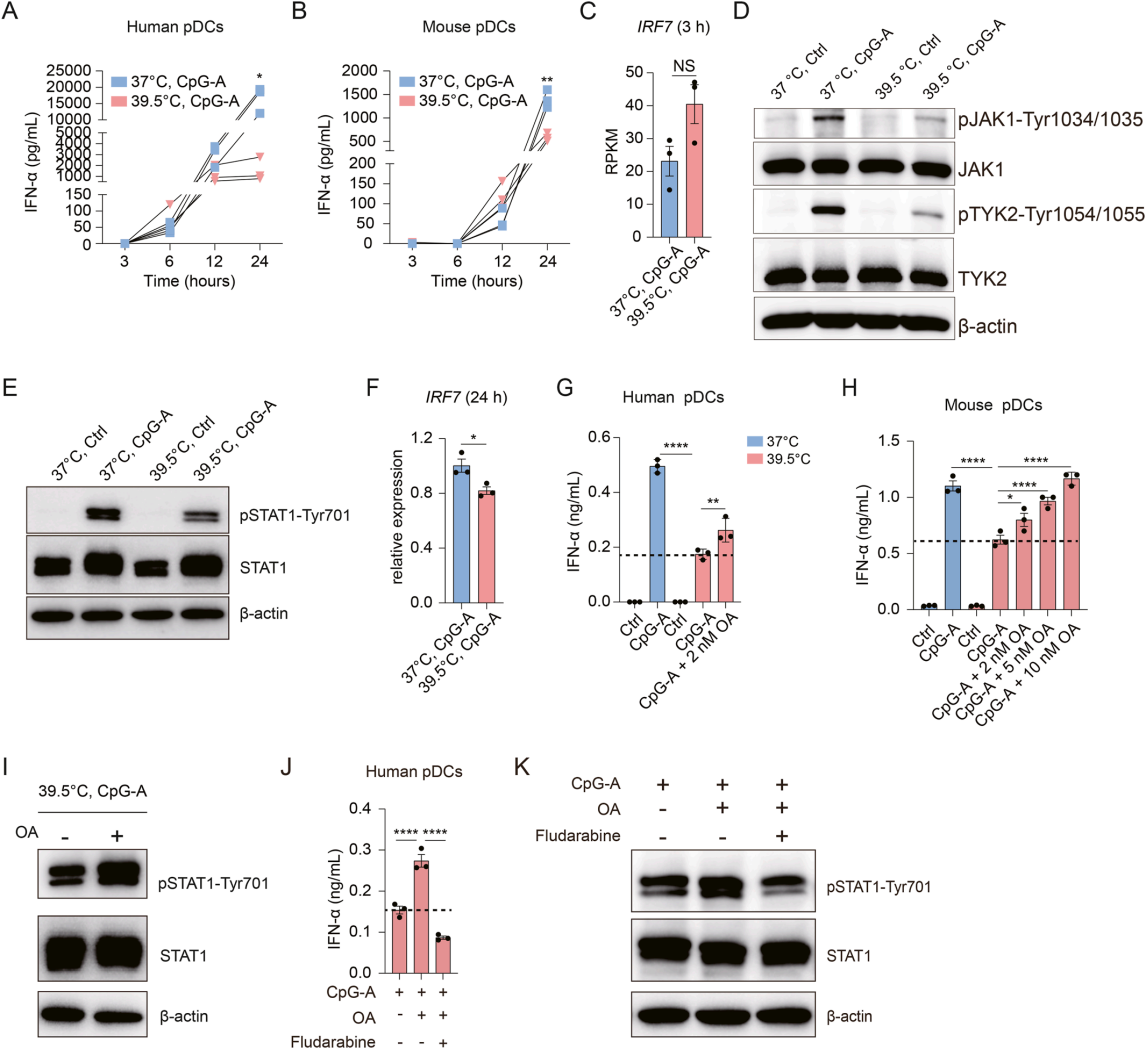
**Febrile temperature suppresses IFN-α production through attenuation of STAT1 phosphorylation.** (A, B) Purified human (A) or mouse (B) pDCs were stimulated with CpG-A at 37 °C or 39.5 °C, and IFN-α production at the indicated time points post stimulation was measured by ELISA. (C) Purified human pDCs were stimulated with CpG-A at 37 °C or 39.5 °C for 3 h, and the expression of *IRF7* was determined by RNA sequencing. (D) Purified human pDCs were cultured with or without CpG-A (1 μM) at 37 °C or 39.5 °C for 9 h. Phosphorylated and total JAK1 and TYK2 protein levels were analyzed by immunoblotting. β-actin served as a loading control. (E) Human pDCs were stimulated with or without CpG-A (1 μM) at 37 °C or 39.5 °C for 12 h, followed by analysis of total STAT1 and phosphorylated STAT1 (Tyr701) by western blotting. β-actin was used as a loading control. (F) Human pDCs were stimulated with CpG-A (1 μM) at 37 °C or 39.5 °C for 24 h. *IRF7* expression were determined by quantitative real-time PCR. (G, H) Human (G) or mouse (H) pDCs were stimulated with or without CpG-A (1 μM) at 37 °C or 39.5 °C for 24 h in the presence or absence of okadaic acid (OA), and IFN-α levels in culture supernatants were assessed by ELISA. (I) Human pDCs were stimulated with CpG-A (1 μM) at 39.5 °C in the presence or absence of OA (2 nM) for 12 h, followed by analysis of total STAT1 and phosphorylated STAT1 (Tyr701) by western blotting. β-actin was used as a loading control. (J, K) Human pDCs were pretreated with or without fludarabine (1 μM) for 6 h and then stimulated with CpG-A (1 μM) at 39.5 °C for 24 h in the presence or absence of OA (2 nM). IFN-α concentrations in culture supernatants were measured by ELISA (J). Total STAT1 and phospho-STAT1 (Tyr701) levels were analyzed by immunoblotting, with β-actin serving as a loading control (K). The control group IFN-α levels in (G) were below the ELISA limit of detection. All experiments were repeated at least three times. Data are presented as mean ± SEM (C, F, G, H, and J). Data in (A, F, G, and J) are representative of three donors. Statistical significance was determined by an unpaired Student's *t*-test (A, B, C, and F) or one-way ANOVA (G, H, and J). ∗*p* < 0.05, ∗∗*p* < 0.01, ∗∗∗*p* < 0.001, ∗∗∗∗*p* < 0.000 1.

Having excluded a major contribution of metabolic pathways, we next focused on identifying the intrinsic signaling node through which febrile temperature constrains IFN-α production in pDCs. We found that CpG-A–induced IFN-α production by pDCs was comparable under normothermic and febrile conditions at 6–12 h post-stimulation but was significantly reduced at 24 h under febrile conditions (Fig. 4(A) and (B)). Consistently, the expression of IRF7, the master regulator of IFN-α production in pDCs, showed no decrease and instead exhibited an upward trend at 3 h under febrile conditions (Fig. 4(C)). These findings suggest that exposure to febrile temperature does not impair early IFN-α induction but instead disrupts the IFN-I–mediated positive feedback loop initiated by early IFN-α production following CpG-A stimulation, thereby limiting sustained IFN-α output from pDCs.

Consistent with this notion, our transcriptomic analysis revealed significant suppression of the JAK–STAT signaling pathway in CpG-A–stimulated pDCs cultured at 39.5 °C for 24 h (Fig. 2(F)). We therefore examined key components of the IFN-I signaling cascade. Western blot analysis showed that phosphorylation of JAK1 and TYK2 was markedly reduced in CpG-A–stimulated pDCs cultured at 39.5 °C compared with those cultured at 37 °C (Fig. 4(D)). Correspondingly, phosphorylation of STAT1 at Tyr701, a major downstream target of JAK1 and TYK2, was also significantly decreased under febrile conditions (Fig. 4(E)). In contrast, total protein levels of JAK1, TYK2, and STAT1 remained unchanged between temperature conditions (Fig. 4(D) and (E)). Moreover, the expression of *TLR9*, *IFNAR1*, and *IFNAR2* was comparable between the two groups (Fig. S3), indicating that febrile temperature suppresses IFN-I signaling primarily through impaired activation of the JAK1/TYK2–STAT1 pathway rather than through reduced expression of these signaling components. Consistent with diminished STAT1 signaling, *IRF7* expression was significantly reduced at 24 h under febrile conditions (Fig. 4(F)), supporting the notion that elevated temperature disrupts the IFN-I positive feedback loop required for sustained IFN-α production.

Given the critical role of STAT1 phosphorylation in IFN-I signal amplification (Ivashkiv and Donlin, 2014), we treated pDCs with the protein phosphatase inhibitor okadaic acid (OA) (Haystead et al., 1989; Takai et al., 1992) during stimulation at 39.5 °C. In human pDCs, 2 nM OA partially restored IFN-α production under febrile conditions (Fig. 4(G)). Similarly, 2 nM OA partially restored IFN-α production in mouse pDCs, while higher concentrations produced a greater restorative effect, with 10 nM OA recovering IFN-α levels to those observed under normothermic conditions (Fig. 4(H)). Western blot analysis showed that OA treatment enhanced STAT1 Tyr701 phosphorylation under febrile conditions (Fig. 4(I)). To determine whether the restorative effect of OA was dependent on STAT1 phosphorylation, we performed a reverse validation experiment using the STAT1 inhibitor fludarabine. Previous studies have shown that 1 μM fludarabine effectively inhibits STAT1 phosphorylation (Xiu et al., 2021). Human pDCs were pretreated with fludarabine for 6 h and then stimulated with CpG-A in the presence of OA at 39.5 °C for 24 h. While OA significantly restored CpG-A-induced IFN-α production under febrile conditions, fludarabine markedly attenuated this rescue effect, reducing IFN-α secretion to levels below those observed with CpG-A stimulation alone (Fig. 4(J)). Consistent with these functional data, OA-induced recovery of STAT1 Tyr701 phosphorylation was substantially diminished by fludarabine treatment (Fig. 4(K)).

Together, these findings indicate that restoration of STAT1 phosphorylation is required for OA-mediated recovery of IFN-α production and support a critical role for the JAK1/TYK2–STAT1 signaling axis in sustaining IFN-α responses under febrile conditions.

## 3 Discussion

Fever is a hallmark of infection and inflammation and has long been appreciated as an evolutionarily conserved host defense mechanism. While its antimicrobial and immunostimulatory roles are well established, how fever shapes specific immune effector programs to balance antiviral protection and immunopathology remains incompletely understood (Evans et al., 2015). In this study, we identify febrile-range temperature as a physiological regulator that selectively constrains pDC–derived IFN-I responses. We demonstrate that elevated temperature suppresses sustained, but not early, IFN-α production by human and mouse pDCs, thereby uncoupling initial pathogen sensing from prolonged interferon amplification. Mechanistically, this effect is mediated by attenuation of STAT1 phosphorylation, resulting in disruption of the IFN-I–dependent positive feedback loop required for robust and sustained IFN-α output.

Our transcriptomic analyses further support this model by revealing broad suppression of IFN-I–associated gene programs, including interferon-stimulated genes and JAK–STAT signaling pathways, specifically in CpG-A–activated pDCs cultured at febrile temperature. In contrast, unstimulated pDCs showed minimal transcriptomic divergence between normothermic and febrile conditions, underscoring the context-dependent nature of thermal regulation. These findings suggest that elevated temperature does not globally dampen pDC function but selectively rewires activated signaling networks downstream of IFN-I, thereby imposing a brake on interferon amplification.

A key finding of our study is that febrile temperature does not impair the early induction phase of IFN-α production in pDCs following TLR9 engagement. Early IFN-α secretion and IRF7 expression were preserved under febrile conditions, indicating that proximal TLR9 signaling and the initial transcriptional activation of IFN-I genes remain intact. Instead, fever selectively limits the late amplification phase of IFN-I production, which is known to rely on autocrine and paracrine IFN-I signaling through the JAK–STAT1–IRF7 axis (Reizis, 2019; Ivashkiv and Donlin, 2014; Marié et al., 1998). This temporal dissociation highlights a previously unrecognized layer of physiological control, whereby fever preserves early antiviral alarm signals while preventing excessive or prolonged interferon exposure.

At the mechanistic level, we identify STAT1 phosphorylation as a central temperature-sensitive node controlling sustained IFN-α production. Febrile temperature markedly reduced STAT1 Tyr701 phosphorylation in CpG-A–stimulated pDCs, leading to diminished expression of IRF7 at later time points. Restoration of STAT1 phosphorylation using the protein phosphatase inhibitor OA was sufficient to rescue IFN-α production in both human and mouse pDCs under febrile conditions, establishing a causal link between impaired STAT1 activation and thermal suppression of IFN-I output.

These findings have important implications for understanding the dual nature of fever in antiviral immunity. While early IFN-I responses are essential for viral control, sustained or excessive IFN-I signaling has been implicated in immunopathology, chronic inflammation, and immune exhaustion in settings such as persistent viral infection and autoimmunity (Cheng et al., 2017; Panda et al., 2017; Barrat and Su, 2019). By selectively limiting prolonged IFN-α production from pDCs, fever may function as a built-in negative feedback mechanism to prevent persistent inflammation and collateral tissue damage. This model supports the idea that fever contributes not only to immune activation but also to immune resolution.

In summary, we demonstrate that fever acts as a conserved physiological regulator that selectively constrains pDC-derived IFN-I responses by disrupting STAT1-dependent positive feedback. These findings suggest a previously unappreciated mechanism by which elevated temperature fine-tunes antiviral immunity and highlight fever as an active immunomodulatory signal rather than a passive byproduct of inflammation. Understanding how physiological cues such as temperature shape immune signaling networks may provide new insights into host defense and inform therapeutic strategies aimed at modulating interferon responses in infectious and inflammatory diseases.

## 4 Materials and methods

### 4.1 Human participants

Human spleen tissues were obtained from trauma patients undergoing splenectomy. All donors provided informed consent following a comprehensive explanation of the study's purpose and implications. The study protocol was approved by the Human Research Ethics Committee of Xuzhou Mine Hospital (Approval Number: XZKSYY2021-002), and all procedures adhered to ethical standards.

### 4.2 Mice

C57BL/6J mice were purchased from GemPharmatech Co., Ltd. All the mice were bred and maintained in Animal Center of Medical Research Institute at Wuhan University. All animal experiments were performed according to the protocols approved by the Animal Care and Use Committee of Medical Research Institute, Wuhan University.

### 4.3 Isolation of human splenocytes

Post-splenectomy specimens were immediately transferred into RPMI-1640 medium (Sigma-Aldrich, R6504) supplemented with 10% fetal bovine serum (FBS; Biological Industries, 04-001-1A) and 1% penicillin–streptomycin (Hyclone, SV30010), and maintained at 4 °C until processing. Tissue processing commenced within 12–24 h of collection. Spleen samples were minced using ophthalmic scissors and subjected to enzymatic dissociation in RPMI-1640 containing collagenase IV (2 mg/mL; BioFRoXX, 2091GR001) and DNase I (0.1 mg/mL; Roche, 10104159001) at a ratio of 5 g tissue per 20 mL solution. Digestion was performed for 20 min at room temperature and terminated by adding 5 mM EDTA (Solarbio, E1170) for an additional 5 min. The resulting cell mixture was passed through a 70-μm cell strainer to remove residual tissue debris. Splenic mononuclear cells were subsequently enriched via Ficoll-Paque Plus density gradient centrifugation (Cytiva, 17-1440-03) and washed with PBS containing 1% FBS and 1 mM EDTA. This workflow enables the reliable recovery of viable splenocytes suitable for downstream analyses.

### 4.4 Isolation of plasmacytoid dendritic cells

Human plasmacytoid dendritic cells were isolated using EasySep™ Human Plasmacytoid DC Isolation Kit (STEMCELL, 17977) from peripheral blood mononuclear cells (PBMCs).

Splenocyte suspensions in PBS supplemented with 1% FBS, and then incubated with Human TruStain FcX (BioLegend, 422302) for 10 min to avoid nonspecific binding. Cells were subsequently incubated with a biotinylated mouse anti-human lineage (lin) antibody cocktail—comprising CD3 (BioLegend, 344820), CD14 (BioLegend, 367106), and CD19 (BioLegend, 302204)—for 15 min at 4 °C. Then cells were labeled with anti-biotin microbeads (Miltenyi Biotec, 130090485) for 15 min at 4 °C. The labeled cells were then depleted using the Mitenyi MidiMACS™ Separator and the unlabeled fraction were collected. The cells were washed, and first incubated with LIVE/DEAD Fixable Aqua Dead Cell Stain (Invitrogen, Cat# L34966) for 10 min. And then the cells were washed and stained with FITC-anti-CD304 (BioLegend, 354512), Percpcy5.5-anti-CD4 (BioLegend, 300530), and PE-anti-CD123 (BioLegend, 306006) antibodies for 30 min at 4 °C. The plasmacytoid dendritic cells (Lin^−^CD4^+^CD123^+^ or Lin^−^CD304^+^CD123^+^) were then sorted using a BD FACS Aria cell sorter (BD Biosciences) or SONY MA900 sorter.

Mouse plasmacytoid dendritic cells were isolated using Mouse Plasmacytoid DC Isolation Kit (Miltenyi Biotec, 130-107-093) from splenocytes.

Bone marrow cell suspensions prepared in PBS containing 1% FBS were initially incubated with LIVE/DEAD Fixable Aqua viability dye for 10 min. After washing, cells were treated with Mouse TruStain FcX (BioLegend, 101320) for 10 min to minimize nonspecific Fc-mediated binding. The samples were then stained with an anti-lineage antibody mixture—CD3 (BioLegend, 100236), CD19 (BioLegend, 152410), and NK1.1 (BioLegend, 108710), all conjugated to APC—along with FITC-anti-CD317 (BioLegend, 127008) and PE-anti-Siglec-H (BioLegend, 129606) for 30 min at 4 °C. Plasmacytoid dendritic cells (Lin^−^CD317^+^Siglec-H^+^) were subsequently isolated using a SONY MA900 cell sorter.

### 4.5 Cells culture and stimulation

Human splenocytes, PBMCs, as well as mouse splenocytes and bone marrow cells were seeded at a density of 5 × 10^5^ cells per well in a 96-well plate and stimulated with or without CpG-A (1 μM, invivogen, tlrl-2216 or tlrl-1585) for 3, 6, 12 or 24 h at either 37 °C or 39.5 °C in complete medium, then the supernatants were collected for ELISA to assess IFN-α and IL-6 secretion.

Cells were stimulated at either 37 °C or 39.5 °C with CpG-A alone, CpG-A plus Ruthenium Red (pan TRPV inhibitor, MCE, HY-103311), CpG-A plus HSP70 inhibitor (Selleckchem, S7751), CpG-A plus prednisolone (MCE, HY-17463), CpG-A plus ketoconazole (MCE, HY-B0105), CpG-A plus tamoxifen (Sigma-Aldrich, T5648-1G), CpG-A plus ZLN005 (MCE, HY-17538), CpG-A plus rosiglitazone (MCE, HY-17386), CpG-A plus okadaic acid (Tocris, 1136) or CpG-A plus fludarabine (MCE, HY-B0069). The supernatants were collected for ELISA to assess IFN-α and IL-6 secretion. Human plasmacytoid dendritic cells were used for cell viability assays, surface staining or RNA-seq.

### 4.6 Surface staining and flow cytometry

For surface staining, human pDCs were washed twice with PBS containing 1% FBS. The cells were first incubated with LIVE/DEAD Fixable Aqua Dead Cell Stain for 10 min, followed by incubation with an Fc receptor–blocking antibody to reduce nonspecific binding. Surface marker staining was performed by incubating cells with FITC-anti-CD303 (BioLegend, 354208) antibody for 30 min at 4 °C. After staining, cells were washed and resuspended in 1% paraformaldehyde for acquisition on a BD LSRFortessa X-20 flow cytometer (BD Biosciences). Data were analyzed using FlowJo software (v10.8.1, BD Biosciences).

### 4.7 RNA isolation and real-time PCR

Total RNA was isolated using RNAiso Plus (TakaRa, 9109) according to manufacturer's instructions. Real-time PCR was performed with the Hieff® qPCR SYBR Green Master Mix (YEASEN, 11198ES08) on a CFX96 system (BIO-RAD, 1855484). The expression of target mRNAs was normalized with β-actin. The fold changes were calculated using 2^−ΔΔCT^ method. The primer sequences were as follows: Human *IRF7*: 5′-GCTGGACGTGACCATCATGTA-3′ (forward) and 5′-GGGCCGTATAGGAACGTGC-3′ (reverse); Human *TLR9*: 5′-GAAGGGACCTCGAGTGTGAA-3′ (forward) and 5′-GTGCTGCCATGGAGAAGTG-3′ (reverse); Human *IFNAR1*: 5′-CGCCTGTGATCCAGGATTATCC-3′ (forward) and 5′-. TGGTGTGTGCTCTGGCTTTCAC-3′ (reverse); Human *IFNAR2*: 5′- ACCGTCCTAGAAGGATTCAGCG-3′ (forward) and 5′- CCAACAATCTCAAACTCTGGTGG-3′ (reverse).

### 4.8 Enzyme-linked immunosorbent assay

For the ELISA bioassay, all measurements were performed using commercial kits according to the manufacturers’ instructions. Absorbance values were calculated using the corresponding standard curves after subtraction of background readings.

### 4.9 *Ex vivo* experiments

Mice were treated with ambient temperature (AT) or fever-range whole-body hyperthermia (WBH) for 20 h and then were sacrificed (Lin et al., 2019; Lin et al., 2021; Chen et al., 2006). Then pDCs were isolated from the spleen. pDCs were stimulated with CpG-A (1 μM) at 37 °C for 24 h. The supernatants were collected for ELISA to assess IFN-α and IL-6 secretion. The cells were used for real-time PCR.

### 4.10 Western blotting

The sorted pDCs were collected and lysed for 20 min in RIPA buffer (50 mM Tris-HCl pH 7.4, 150 mM NaCl, 1% NP-40, 1 mM EDTA) supplemented with protease and phosphatase inhibitors. Protein extracts were then mixed with loading buffer, boiled at 95 °C for 5 min, separated by SDS-PAGE on 8% or 10% gels, and transferred to nitrocellulose membranes. The membranes were blocked with 5% BSA in TBST and then incubated overnight at 4 °C with primary antibodies against phospho-STAT1 (Tyr701, CST, 9167), total STAT1 (CST, 14994), phospho-TYK2 (Tyr1054/1055, CST, 68790), total TYK2 (CST, 14193), phospho-JAK1 (Tyr1034/1035, CST, 74129), JAK1 (CST, 50996) and β-actin (Santa Cruz Biotechnology, sc-47778). After washing with TBST, the membranes were incubated with appropriate HRP-conjugated goat anti-rabbit IgG (ABclonal, AS039) or HRP-conjugated goat anti-mouse IgG (Proteintech, SA00001-1) at room temperature. After washing, bound antibodies were detected using Pierce ECL Plus Western Blotting Substrate (Thermo, 32106), and chemiluminescent signals were captured with a ChemiDoc™ Imaging System (BIO-RAD, 12003153).

### 4.11 Bulk RNA-sequencing data processing

The sorted pDCs from human splenocytes were cultured at 37 °C or 39.5 °C and stimulated with or without CpG-A for 3 h or 24 h. The pDCs were collected in TRIzol for RNA extraction. Raw sequencing data was first filtered by Trimmomatic (version 0.36), low-quality reads were discarded and the reads contaminated with adaptor sequences were trimmed. Clean Reads were further treated with in-house scripts to eliminate duplication bias introduced in library preparation and sequencing. In brief, clean reads were first clustered according to the UMI sequences, in which reads with the same UMI sequence were grouped into the same cluster. Reads in the same cluster were compared to each other by pairwise alignment, and then reads with sequence identity over 95% were extracted to a new sub-cluster. After all sub-clusters were generated, multiple sequence alignment was performed to get one consensus sequence for each sub-cluster. After these steps, any errors and biases introduced by PCR amplification or sequencing were eliminated.

The de-duplicated consensus sequences were used for standard RNA-seq analysis. They were mapped to the human reference genome using STAR software (version 2.7.10a) with default parameters. Reads mapped to the exon regions of each gene were counted by feature Counts (Subread-1.5.1; Bioconductor).

### 4.12 Statistics analysis

All experimental data were analyzed using GraphPad Prism version 10.1.0 (GraphPad Software, San Diego, CA). Results were presented as mean ± SEM. Differences between groups were assessed using two-tailed unpaired Student's *t*-tests, one-way ANOVA followed by Dunnett's multiple comparisons test or two-way ANOVA followed by Tukey's multiple comparisons test. A *p* value of less than 0.05 was considered statistically significant. Statistical significance is indicated in graphs as follows: ∗*p* < 0.05, ∗∗*p* < 0.01, ∗∗∗*p* < 0.001, and ∗∗∗∗*p* < 0.000 1. NS, not significant.

## CRediT authorship contribution statement

**Yangyang Xu:** Writing – original draft, Visualization, Methodology, Investigation. **Hao Yang:** Visualization, Methodology, Investigation. **Lin Yang:** Investigation. **Qing Zhang:** Investigation. **Liang Cheng:** Writing – review & editing, Writing – original draft, Visualization, Project administration, Methodology, Investigation, Funding acquisition, Conceptualization.

## Data availability

All data generated or analyzed during this study are included in the main text or the Supplementary Material.

## Declaration of competing interest

The authors declare that they have no known competing financial interests or personal relationships that could have appeared to influence the work reported in this paper.

## Appendix A Supplementary data

Multimedia component 1

Table S1 Comparison of pDCs under febrile (39.5°C) and normothermic (37°C) conditions, related to Fig. 2.
