# Supplementary material for: Febrile-range temperature selectively limits sustained pDC-Derived IFN-α via attenuation of STAT1-Dependent feedback signaling
Source: Cell Insight. 2026 Jun 24;5(5):100344. doi: 10.1016/j.cellin.2026.100344 (PMC13445209; doi:10.1016/j.cellin.2026.100344)
Supplement: Supplementary file 1 [file mmc1.docx]

**Supplemental Figures 1-3**

**Febrile-Range Temperature Selectively Limits Sustained pDC-Derived IFN-α via Attenuation of STAT1-Dependent Feedback Signaling**

Yangyang Xu^a,b^, Hao Yang^a,b^, Lin Yang^c^, Qing Zhang^d^, Liang Cheng^a,b,*^

**Affiliations:**

^a^ Department of Infectious Diseases, Medical Research Institute, Frontier Science Center for Immunology and Metabolism, State Key Laboratory of Virology and Biosafety, Zhongnan Hospital of Wuhan University, Wuhan University, Wuhan 430071, Hubei, China

^b^ Department of Otolaryngology, Head and Neck Surgery, Zhongnan Hospital of Wuhan University, Wuhan University, Wuhan 430071, Hubei, China

^c^ Department of General Surgery, General Hospital of Xuzhou Mining Group, Xuzhou 221000, Jiangsu, China

^d^ Cancer Institute, Xuzhou Medical University, Xuzhou, 221004, Jiangsu, China

*Correspondence: liangcheng@whu.edu.cn (L. Cheng)

**Figure S1. Temperature-dependent inhibition of IFN-α production**

**(A–C)** Human PBMCs were stimulated with CpG-A (1 μM) at 37°C, 38.5°C, or 39.5°C for 24 hours, and IFN-α levels in culture supernatants were measured by ELISA.

Data are representative of three independent experiments and are presented as mean ± SEM. Statistical significance was determined using one-way ANOVA followed by Tukey’s multiple comparisons test (A–C). **p* < 0.05, ***p* < 0.01, ****p* < 0.001, *****p* < 0.0001.

**Figure S2. HSP70 Inhibition Does Not Impair pDC Viability Under Febrile Temperature**

**(A, B)** Human PBMCs were cultured with or without CpG-A (1 μM) at 37°C or 39.5°C for 24 hours in the presence or absence of the indicated HSP70 inhibitor. pDC viability was assessed by flow cytometry. Representative flow cytometry plots are shown in (A), and quantitative analysis is presented in (B).

Data are presented as mean ± SEM (B) and are representative of three independent donors.

**Figure S3. Febrile Temperature Does Not Alter the Expression of *TLR9* and Type I Interferon Receptors in Human pDCs**

**(A)** Purified human pDCs were stimulated with CpG-A (1 µM) at 37°C or 39.5°C. *TLR9* expression was determined by RNA sequencing after 3 hours of stimulation (left), and by quantitative real-time PCR after 24 hours (right).

**(B–D)** Purified human pDCs were stimulated with CpG-A (1 μM) at 37°C or 39.5°C. IFNAR1 and IFNAR2 expression was assessed by RNA sequencing (B) or quantitative real-time PCR (C, D) at the indicated time points.

Data are presented as mean ± SEM (A–D). Statistical significance was determined using unpaired Student’s t-test (A–D). **p* < 0.05, ***p* < 0.01; NS, not significant.
